# Safety and efficacy of physical activity in hypertrophic cardiomyopathy patients: a systematic review and metanalysis

**DOI:** 10.3389/fcvm.2025.1739956

**Published:** 2026-03-12

**Authors:** Francesco Borrelli, Filippo Tarditi, Alessandro Andreis, Barbara Mabritto, Andrea Silvio Benso, Elisabetta Toso

**Affiliations:** 1 Postgraduate School of Sports and Exercise Medicine, Department of Medical Science, University of Turin, Turin, Italy; 2 Advanced Cardiovascular Echocardiography Unit, Department of Cardiovascular and Thoracic Sciences, Città della Salute e della Scienza, Turin, Italy; 3 Division of Cardiology, Ordine Mauriziano Hospital, Turin, Italy; 4 Division of Endocrinology, Diabetology and Metabolism, Department of Medical Sciences, University of Turin, Turin, Italy

**Keywords:** exercise, exercise prescription, HCM, low risk cardiomyopathy, safety, SCD, VO_2_max

## Abstract

Hypertrophic Cardiomyopathy (HCM) is a fairly common inherited cardiac disease, with a prevalence of about 1:200–1:500, characterized by left ventricular hypertrophy (LVH ≥15 mm) often associated with microvascular dysfunction, myocardial fibrosis and major ventricular arrhythmic events. Historically, HCM has been managed conservatively, with universal restrictions on vigorous physical activity due to concerns about ventricular arrhythmias and sudden cardiac death (SCD), particularly in the context of competitive sports. These recommendations, once codified in the 2020 AHA and ESC guidelines, led to significant secondary consequences including increased risk of obesity, metabolic syndrome, and psychological distress. The 2023 Italian Cardiology Protocols for Eligibility for Competitive Sport (COCIS), aligned with the European and American guidelines, provide a rigorous diagnostic framework for assessing risk in individuals with HCM. Although these protocols outline criteria for competitive sports eligibility, the evidence reviewed in this meta-analysis predominantly derives from studies investigating patients engaged in recreational or moderate-intensity exercise rather than competitive athletes. Therefore, the applicability of current findings to competitive sports participation remains limited and should be interpreted with caution. Recent evidence challenges the one-size-fits-all approach, emphasizing the therapeutic benefits and safety of regular moderate-intensity exercise in individuals with low-risk HCM. This paradigm shift is reflected in updated international guidelines, including the 2023 ESC and 2024 AHA/ACC recommendations, which now recognize that universal restriction from vigorous exercise is not warranted for most patients with HCM. These documents advocate for a more nuanced, individualized approach based on shared decision-making and comprehensive evaluation through cardiopulmonary exercise testing (CPET), exercise echocardiography, and longitudinal follow-up. The evolving role of exercise in HCM highlights the need for structured, personalized prescriptions that consider arrhythmic risk stratification and patient preferences and goals. The present work aims to critically synthesize contemporary evidence regarding HCM and physical activity, with particular focus on the incidence of major adverse cardiovascular events (MACE) in athletic populations, and the implications for future clinical management. The meta-analysis included 8 studies on 2217 patients with HCM, of whom 1204 were in the exercise group and 1013 in the control group. The results demonstrate that exercise is safe and does not increase the risk of MACE (RR 1.01, *p* = 0.97) and is effective in improving cardiorespiratory fitness (+1.76 ml/kg/min in VO2 peak, *p* < 0.0001). These results support the inclusion of structured and supervised exercise in the clinical management of patients with HCM.

## Introduction

Hypertrophic cardiomyopathy (HCM) is one of the most prevalent inherited structural heart diseases, with an estimated prevalence of 1:500 in the general population (1–5). Characterized by unexplained left ventricular hypertrophy, HCM presents a broad phenotypic and prognostic spectrum, ranging from asymptomatic forms to severe clinical manifestations, including heart failure, ventricular arrhythmias, and sudden cardiac death (6–12).

Traditionally, international sports cardiology guidelines have recommended restricting vigorous physical activity for patients with HCM, based primarily on observational data suggesting an increased risk of arrhythmic events during vigorous exercise (13–15). Recent studies have reported significantly elevated rates of SCD in high-physiological-stress disciplines such as competitive bodybuilding in both males and females (16, 17). However, this restrictive approach has resulted in significant limitations on patients' quality of life, particularly for young and physically active individuals (18).

In recent years, growing evidence has increasingly challenged this paradigm, suggesting that structured and supervised exercise may not only be safe but also beneficial for patients with HCM (19–23). Several studies have demonstrated improvements in functional capacity (24), quality of life, and cardiometabolic parameters (25, 26), without any corresponding rise in major adverse cardiovascular events (MACE) (10, 14, 18–22, 27–29) also with the use of CPET (30, 31).

In the Italian regulatory framework, eligibility for competitive sports is governed by the COCIS protocols (3). While international guidelines increasingly promote shared decision-making for sport participation, this principle does not apply to competitive sports in Italy, where the final judgment lies exclusively with the sports medicine physician. Shared decision-making is instead fully applicable in determining safe levels of recreational and therapeutic exercise.

Several systematic reviews have previously examined the relationship between exercise and hypertrophic cardiomyopathy, but these analyses have been limited by smaller samples, narrower inclusion criteria, or outdated evidence. For example, the 2025 meta-analysis by Albulushi et al. (32). included fewer randomized trials and did not integrate the most recent high-intensity training trials published in 2024–2025. Moreover, prior reviews focused predominantly on safety outcomes, whereas the present study concurrently evaluates both efficacy (VO₂ peak changes) and safety (MACE incidence), using the most comprehensive dataset to date, updated to May 2025.

Importantly, the studies included in this meta-analysis evaluate individuals involved in recreational, moderate, or supervised structured exercise, not competitive athletic participation, which limits the generalizability of findings to decisions regarding competitive eligibility.

## Objectives

The primary aim of this review is to assess the safety of exercise in patients with HCM by analyzing the incidence of MACE, including sudden cardiac death, resuscitated cardiac arrest, sustained ventricular tachycardia, and appropriate implantable defibrillator shocks.

A secondary objective is to evaluate the efficacy of exercise in improving cardiorespiratory fitness, measured by changes in peak oxygen consumption (VO_2_ peak) between the exercise training group and the control group.

## Methods

### Research strategy and study selection process

A systematic search of literature was conducted in major biomedical databases (PubMed/MEDLINE, Embase, Cochrane Central Register of Controlled Trials) updated to May 2025. The following search strings were used:

- PubMed/MEDLINE: “hypertrophic cardiomyopathy"[MeSH] OR “HCM” AND (“exercise” OR “physical activity” OR “training” OR “rehabilitation”) AND (“clinical trial” OR “cohort” OR “observational study”)
- Embase: ‘hypertrophic cardiomyopathy'/exp AND (‘exercise'/exp OR ‘physical activity’ OR ‘training’) AND (‘clinical trial'/exp OR ‘cohort study'/exp)
- Cochrane CENTRAL: (hypertrophic cardiomyopathy) AND (exercise OR training).

**Figure 1:**
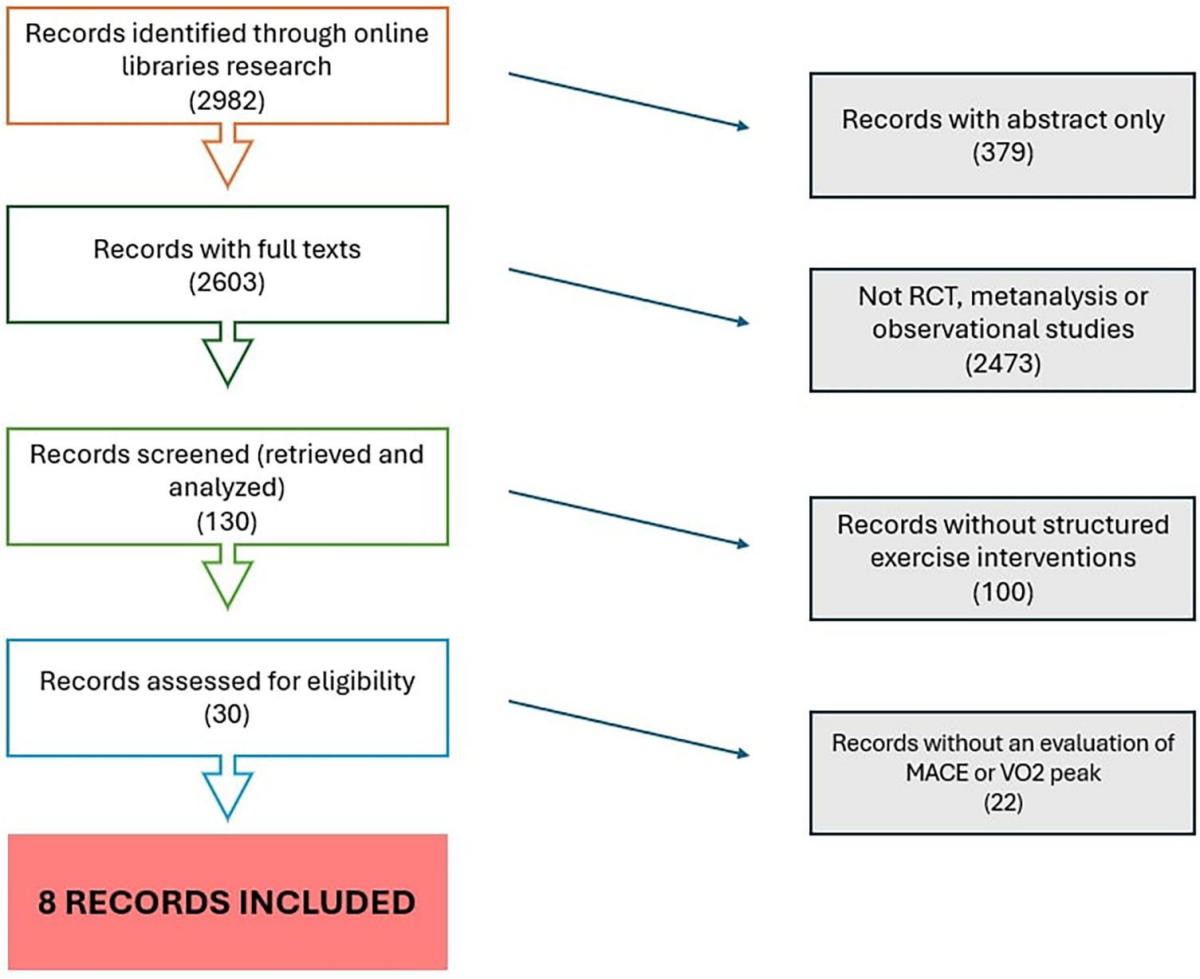
PRISMA flow diagram. PRISMA, preferred reporting items for systematic reviews and meta-analyses.

A comprehensive search strategy was developed according to PRISMA-S guidelines (Figure 1).

Two independent reviewers screened titles, abstracts, and full texts. Disagreements were resolved by consensus or by a third reviewer when necessary. Data extraction was performed independently by the same reviewers using a standardized form.

The protocol was registered on PROSPERO with the ID 1149432 created on the 16 September 2025.

### Inclusion ed exclusion criteria

#### Inclusion criteria

- Randomized controlled trials (RCTs) and observational studies (cohort, case-control)
- Patients with confirmed diagnosis of hypertrophic cardiomyopathy
- Structured exercise interventions of any intensity and duration
- Presence of a control group
- Safety outcomes (MACE events) and/or efficacy outcomes (VO₂ peak) reported

#### Exclusion criteria

- Case reports, case series, editorials, letters
- Studies on pediatric populations (<18 years)
- Patients with secondary hypertrophic cardiomyopathy
- Lack of extractable data for meta-analysis
- Duplication of already included cohorts

### Risk of bias assessment

**Figure 2:**
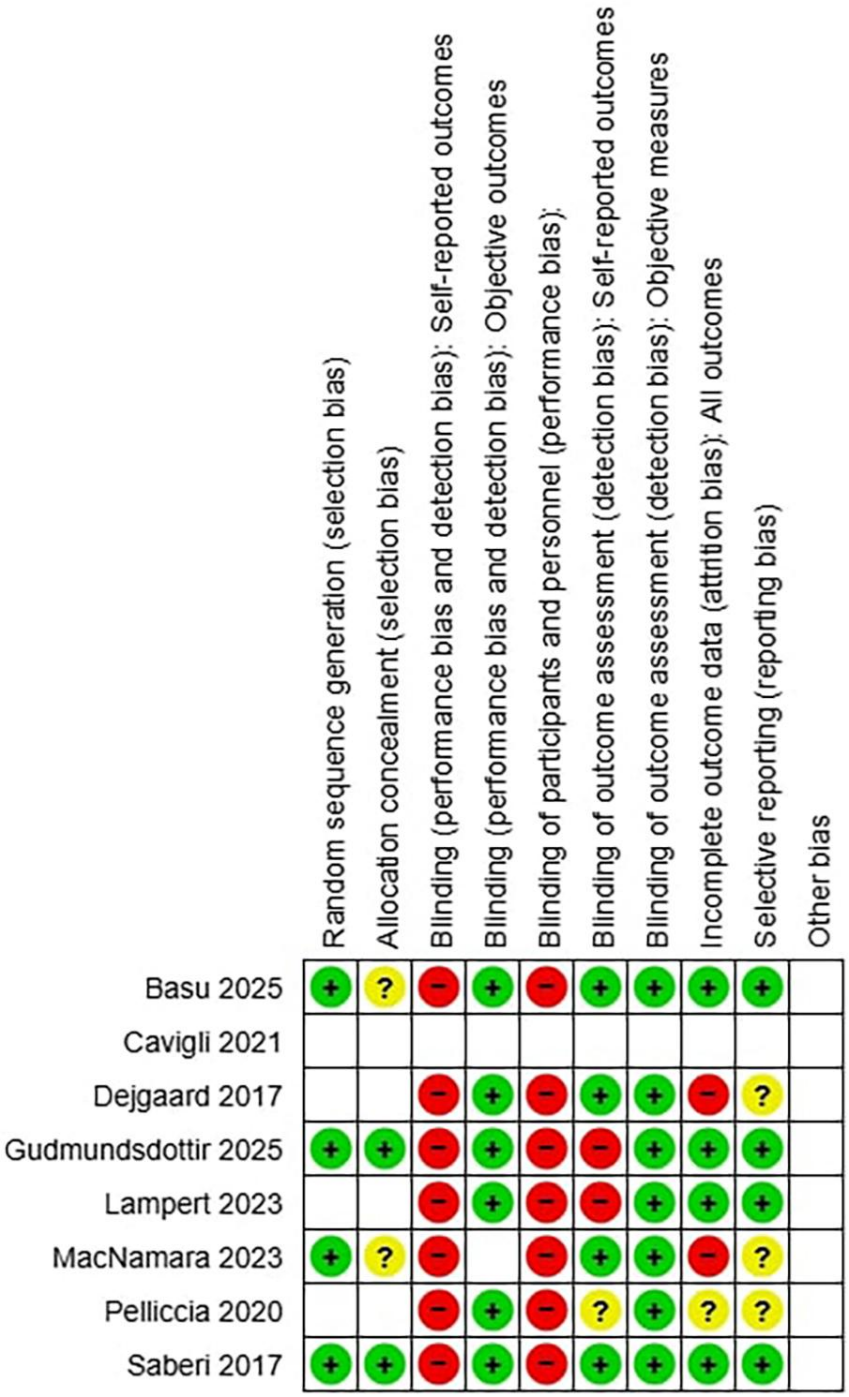
(on the left): types of bias of each study, green+ if the bias is present, red—if the bias is absent, yellow? if the bias is uncertain.

**Figure 3:**
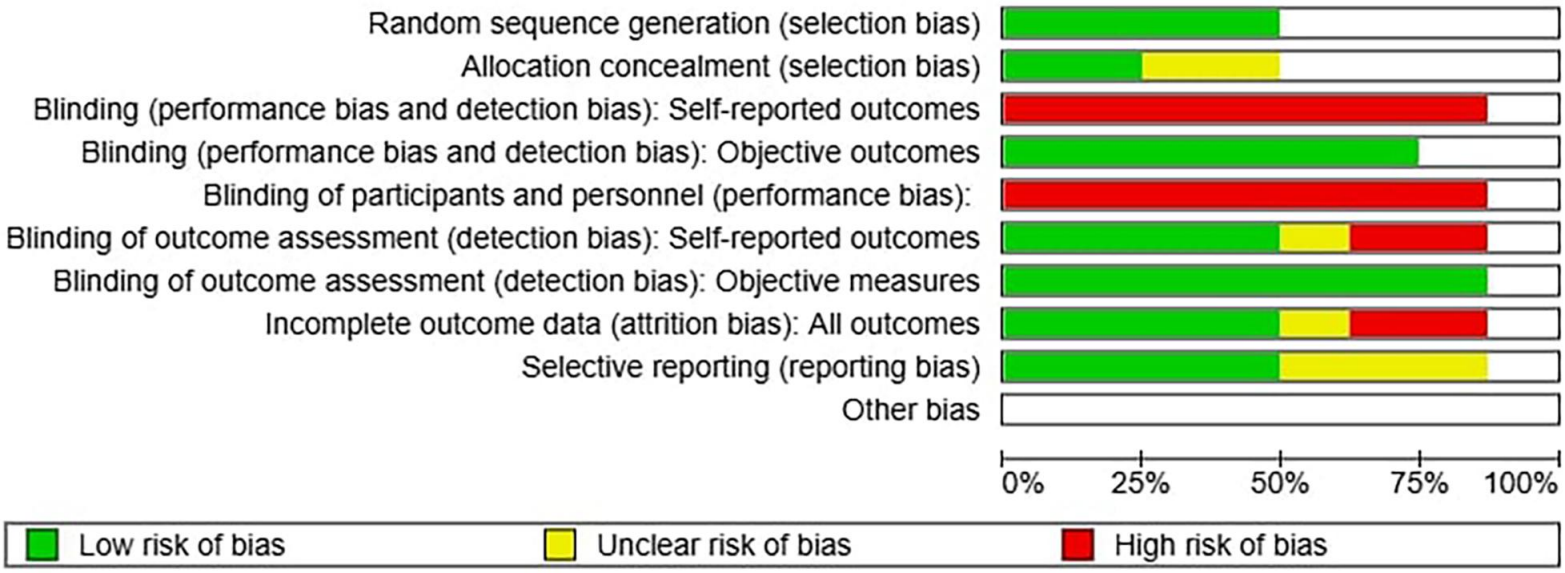
(above): summary of bias considering all studies.

Risk of bias was assessed using validated tools appropriate for each study design. Randomized controlled trials were evaluated with the Cochrane RoB 2 tool, whereas observational studies were assessed using ROBINS-I. Two reviewers independently performed the assessments, with discrepancies resolved through discussion. Risk-of-bias judgments did not influence study weighting in the meta-analysis, which followed methodological conventions of aggregating effect sizes irrespective of quality; however, results were interpreted in light of the identified limitations. Sensitivity analyses excluding studies at high risk of bias were considered but not performed due to the small number of available randomized trials and the consequent loss of statistical power. Risk of bias assessment is summarized in Figures 2, 3.

### Data

The extracted information included:

- Study characteristics: authors, year, design, and follow-up duration
- Population characteristics: sample size, age, sex, HCM phenotype, and presence of left ventricular outflow tract obstruction
- Intervention details: type, intensity, duration, and frequency of exercise
- Primary and secondary outcomes: safety outcomes (MACE events) and efficacy outcomes (VO₂ peak)
- Data for risk of bias assessment: information to evaluate the risk of bias in included studies

### Statistical analysis

The meta-analyses were conducted using Review Manager 5.4 software. For dichotomous outcomes (MACE events), risk ratios (RR) with 95% confidence intervals were calculated, while for continuous outcomes (VO₂ peak), mean differences (MD) or standardized mean differences (SMD) were used when appropriate. Given the clinical heterogeneity among included studies, a random-effects model (DerSimonian–Laird) was chosen *a priori* for all analyses. Zero-event studies were handled using a continuity correction of 0.5 added to each cell of the 2 × 2 table. Heterogeneity was assessed using I² and Cochran's Q, and potential sources of heterogeneity were explored through subgroup analyses (RCTs vs. observational studies, exercise intensity categories). Publication bias was evaluated through visual inspection of funnel plots and, when applicable (≥10 studies), Egger's regression test. Because only eight studies met inclusion criteria, formal statistical testing for funnel-plot asymmetry was interpreted with caution.

### Studies included

The systematic search identified eight eligible studies published between 2017 and 2025, including four randomized controlled trials (RCTs) and four observational studies. The studies enrolled a total of 2,217 patients with hypertrophic cardiomyopathy, with a mean age ranging from 42 to 58 years. The exercise protocols included moderate- to vigorous aerobic training, with durations ranging from 12 to 24 weeks for the RCTs and long-term follow-up evaluations for the observational studies.

## Results

The meta-analysis included eight studies conducted in various geographic and clinical settings. Four randomized controlled trials (7, 29, 33, 34) provided evidence on the efficacy of exercise, while four observational studies (6, 11, 20, 27) primarily contributed to the evaluation of long-term safety, including larger populations and extended follow-up periods (Tables 1, 2). The analysis of safety and efficacy of exercise in patients with hypertrophic cardiomyopathy (HCM) included a total of 2,217 patients, with 1,204 assigned to the exercise group and 1,013 to the control group. The heterogeneity between studies was very low (I^2^ = 0%), suggesting consistency of results across different populations and exercise protocols. In the analysis of the relationship between exercise and major adverse cardiovascular events (MACE) in HCM (Figure 3), the overall risk ratio was 1.01 (95% CI: 0.72–1.41) with a p-value of 0.97, indicating no statistically significant differences between the two groups. Subgroup analysis showed consistent results in both observational studies (RR 0.97, 95% CI: 0.68–1.39) and randomized trials (RR 1.33, 95% CI: 0.50–3.49), although the latter showed wider confidence intervals due to smaller sample sizes. Regarding efficacy analysis, no significant differences were observed in baseline VO₂ peak values between the intervention and control groups (mean difference: −1.09 ml/kg/min, 95% CI: −2.78 to 0.60, *p* = 0.21), confirming the adequacy of randomization in controlled clinical studies (Figure 5). However, at the end of the exercise programs, a statistically significant and clinically relevant improvement was observed in the group undergoing physical training. The mean difference in VO₂ peak change was +1.76 ml/kg/min (95% CI: 0.91–2.61) in favor of the exercise group, with a *p*-value < 0.0001 (Figure 6). This improvement, documented in four high-quality randomized trials (7, 29, 33, 34), represents a clinically significant increase in functional capacity, translating to improved quality of life and exercise tolerance in patients.

**Table 1:** Main features of all included studies.

Author, Year	Type	Inclusione criteria	Exclusion criteria	Outcomes	Experimental treatment design
Saberi et al. 2017 (34)	RCT	HCM, 18–80 yrs	NYHA IV, arrhytmias, effort syncope, CAD, exercise limitations, FE < 55%; pregnancy	VO2 peak	ET (moderate intensity) vs. usual care
Dejgaard et al. 2018 (27)	Retrospective/observational	HCM	myectomy	MACE	Athletes (>4 h/wk >6 years) vs. non atheletes (<4 h/w o <6/years)
Pelliccia et al. 2020 (6)	Retrospective/observational	HCM, regular EF	/	MACE	Continua ET vs. STOP
Lampert et al. 2023 (20)	Prospective/observational	HCM,8–60 years,	NYHA III-IV,syndromic/infiltrative diseases	MACE	Non vigorous ET (sedentary and moderate) vs. vigorous ET
Cavigli et al. 2021 (28)	Prospective/observational	HCM, >18 years	NYHA III-IV, effort symptoms, exercise limtations, recent hospitalitation < 3 mesi, myectomy	VO2 peak	ET vs. STOP
MacNamara et al. 2022 (7)	RCT	HCM	NYHA IV, effort symptoms, LVOT obstruction, pregnancy, myectomy, recent hospitalitation, CAD	vo2peak	HIT vs. MICT
Basu et al. 2025 (29)	RCT	HCM, 16- 60 years, NYHA I e II, able to ex.	athtletes, arrhytmias, syncope, CAD, myectomy, ICD, LVOT obstruction, FE < 35%, renal diseases, pregnancy	MACE	HIT vs. usual care
Gudmundsdottir et al. 2025 (33)	RCT	HCM, >18 yrs	LVOT obstruction, effort symptoms, myectomy, valvulopathy, CAD, exercise limitations, athletes	vo2peak	ET vs. usual care

Tables 1, 2 were revised to standardize inclusion/exclusion criteria, terminology, and formatting. Missing data are uniformly labeled as “Not reported (NR)” instead of dashes. Exercise interventions are described consistently by type, intensity, and duration whenever available.

NYHA, New York Heart Association; LVOT, left ventricle outflow tract; CAD, coronary artery disease; Vo2 Peak, peak oxygen consumption; MACE, major adverse cardiovascular events; ET, exercise training; STOP, stop training; HIT, high intensity training; MICT, moderate intensity continuous training.

**Table 2:** Comparison between training group and control group in term of major adverse cardiovascular events and peak oxygen consumption in all studies included.

Author	N°	Age	GEN (M %)	N° ET	ADV EV. MACE ET	N Contr	ADV EV. MACE Contr.	VO2 peak T0 ET	VO2 peak T1 ET	Delta VO2 ET	VO2 peakT0 Contr	VO2 peak T1 Contr	Delta VO2 Contr
Saberi et al. 2017 (34)	136	50	58	69	0	67	0	21.3 (6.3)	22.6 (6.3)	1.27 (3.5)	22.5 (7.2)	22.5 (7.2)	0.08 (3.5)
Dejgaard et al. 2018 (27)	121	55	53	44	11	77	17	–	–	–	–	–	–
Pelliccia et al. 2020 (6)	88	31	81	27	0	61	2	–	–	–	–	–	–
Lampert et al. 2023 (20)	1,660	39	69	961	44	699	33	–	–	–	–	–	–
Cavigli et al. 2021 (28)	71	38.5	90	33	0	38	5	32.9 (7.4)	–	–	25.2 (7.4)	–	–
MacNamara et al. 2022 (7)	15	47.5/8.7	67	7	1	8	1	25.04 (7.5)	26.56 (8.67)	1.52 (8.15)	23.79 (5.69)	24.89 (6.01)	1.1 (5.86)
Basu et al. 2025 (29)	67	48/44	90/77,5	34	7	33	5	28.3 (8.8)	30.2 (8.7)	1.9 (9.7)	30.3 (10)	28.2 (8.7)	−2.1 (9.7)
Gudmundsdottir et al. 2025 (33)	59	58	73	29	0	30	0	20.2 (6.4)	22 (7.6)	1.8 (2.0)	21.1 (7)	20.8 (9)	−0.3 (3.1)

ADV EV. MACE ET/Contr, adverse events in term of MACE in the exercise group or in the control group; VO2 peak T0/T1 Et/Contr, oxygen peak consumption before training (T0) and after training (T1) in the exercise group (ET) or in the control group (Contr.); Delta Vo2 peak, difference between T1 and T0.

**Figure 4:**
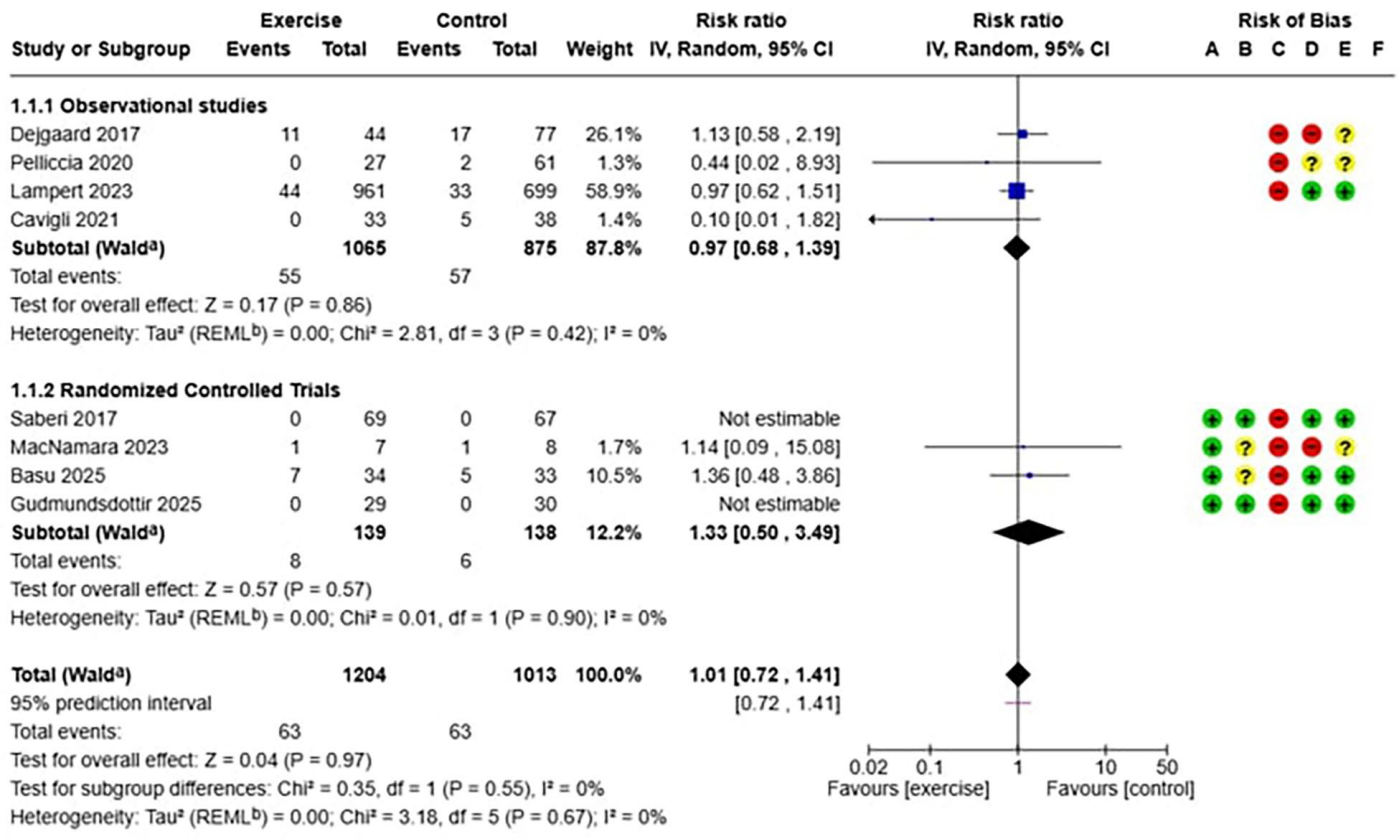
(below): forest plot which shows the relationship between exercise and major adverse cardiovascular events (MACE) in HCM.

**Figure 5:**
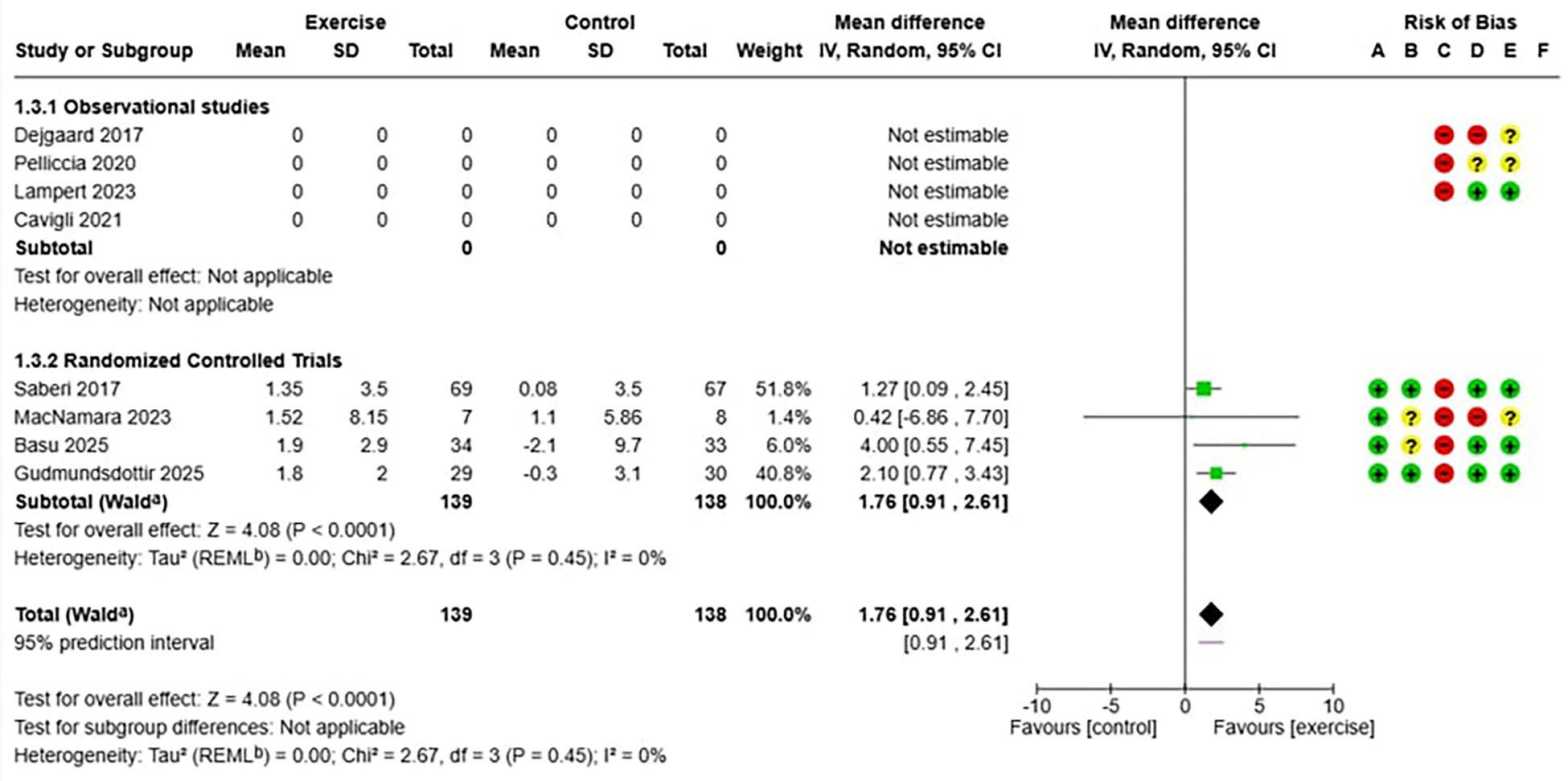
(on the top): forest plot which shows the mean difference in term of peak oxygen consumption (VO_2_ peak) between exercise and control group before the training.

**Figure 6:**
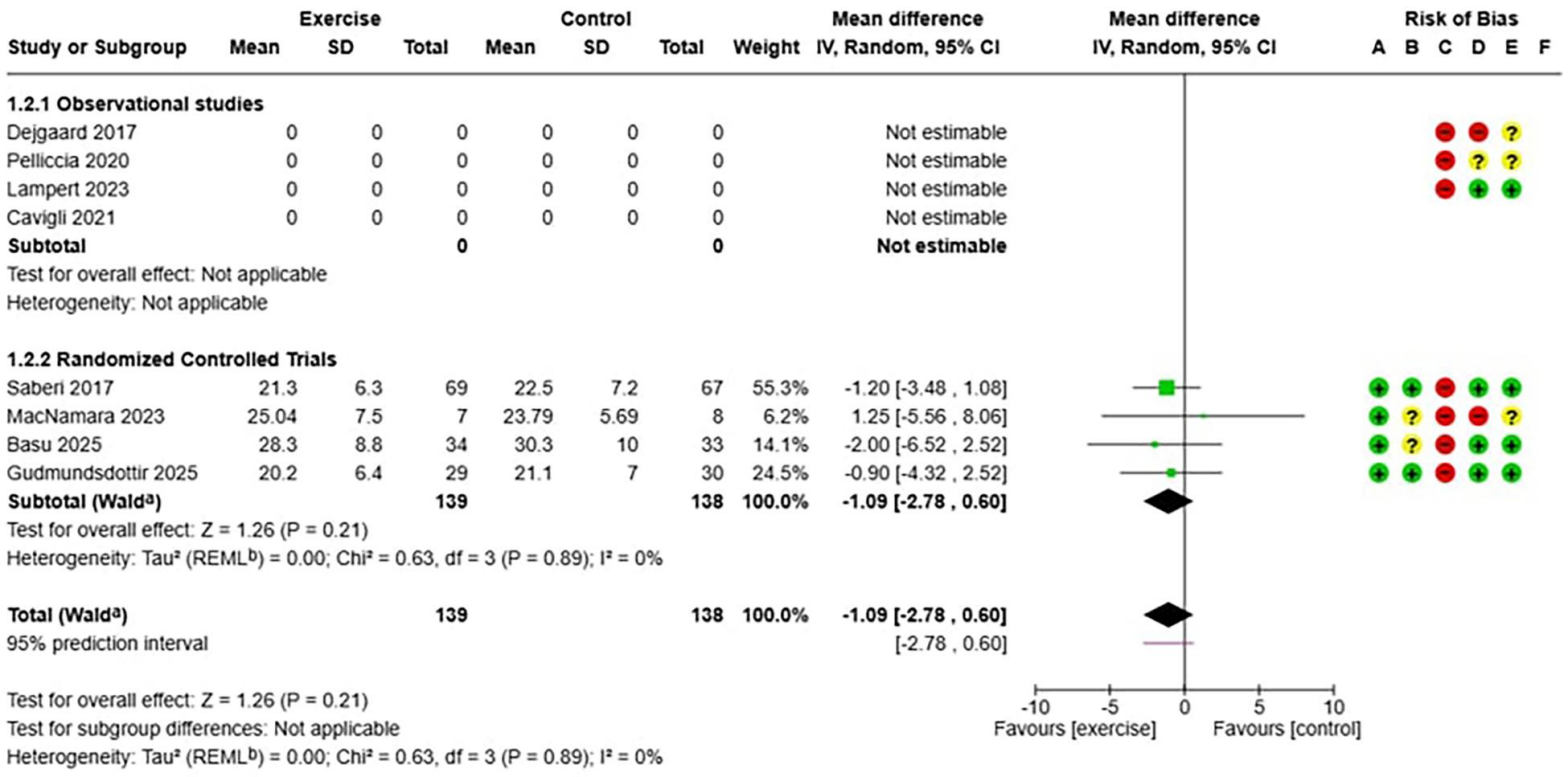
(above): forest plot which shows the mean difference in term of peak oxygen consumption (VO_2_ peak) between exercise and control group after the training.

In conclusion, our meta-analysis provides robust and reassuring evidence on the safety and efficacy of structured exercise in this population. The results demonstrate that exercise does not increase the risk of major adverse cardiovascular events (RR 1.01, 95% CI: 0.72–1.41, *p* = 0.97) and, at the same time, leads to a clinically significant improvement in cardiorespiratory functional capacity (+1.76 ml/kg/min in VO₂ peak, *p* < 0.0001).

## Discussion

A crucial point in interpreting these findings is that the majority of the included studies did not investigate competitive athletes but rather patients engaging in moderate-intensity or supervised structured exercise. Only a minority of studies (21, 23, 27, 29) evaluated individuals performing vigorous training, and even in these cases, competitive participation was not systematically assessed. Therefore, while the results support the safety of exercise in low-risk HCM patients, they cannot be directly extrapolated to high-intensity competitive sports participation.

The most relevant finding from this analysis concerns the cardiovascular safety of exercise in patients with hypertrophic cardiomyopathy (HCM). The absence of a statistically significant increase in MACE incidence represents a clinically important outcome, challenging traditional restrictive recommendations based primarily on outdated case reports. The low heterogeneity observed (I^2^ = 0%) further strengthens this finding, suggesting consistency across different populations, exercise protocols, and clinical settings.

The 1.76 ml/kg/min improvement in VO₂ peak observed in the exercise group represents a clinically significant increase in functional capacity. This improvement, equivalent to approximately 7%–10% compared to baseline values typical of patients with HCM (7, 34) translates into tangible benefits in daily life, including increased tolerance to physical activities, reduced exertional dyspnea, and improved perceived quality of life.

The mechanisms underlying this improvement are multifaceted and involve both central and peripheral adaptations. At the cardiac level, aerobic exercise can lead to improvements in diastolic function, reduced ventricular stiffness, and optimized ventricular filling.

These results emphasize the importance of appropriately structured and supervised exercise programs, preceded by accurate individual risk stratification. This methodological approach reflects optimal clinical practice and highlights the need for specialist evaluation before starting any exercise program.

The prescription of exercise in patients with HCM requires specialized expertise and a multidisciplinary approach. Evaluation should include a thorough family and personal history, physical examination, electrocardiogram, echocardiogram, cardiopulmonary exercise testing (CPET), and, when indicated, Holter monitoring and cardiac magnetic resonance imaging.

The cardiopulmonary exercise test (CPET), CPET, possibly combined with stress echocardiography, is essential for the functional assessment of patients with hypertrophic cardiomyopathy and for individualized exercise prescription. VO₂peak reflects maximal aerobic capacity, while the anaerobic threshold (AT/VT1) delineates the upper limit of sustainable effort and guides the selection of safe training intensities. The second ventilatory threshold (VT2/RCP) identifies the high-intensity domain, to be used only in selected and closely supervised protocols. Parameters such as the VE/VCO₂ slope, ventilatory graphs, and pulse oximetry quantify ventilatory efficiency and help tailor interventions targeting respiratory muscle strength when limitations are present. The O₂ pulse trend supports the detection of left ventricular dysfunction and the definition of thresholds beyond which exercise may become harmful. CPET also enables identification of chronotropic incompetence, abnormal blood pressure responses, and exercise-induced arrhythmias, thereby establishing safe heart rate and blood pressure ranges for training.

While the physiological rationale for exercise benefits is well established, the actual clinical evidence remains limited by short intervention periods, heterogeneous training programs, and incomplete assessment of disease progression. These factors underscore the need for cautious interpretation.

The findings of this meta-analysis support a paradigm shift in the clinical management of patients with HCM, moving from a universally restrictive approach to a personalized strategy based on individual risk stratification. This shift is already reflected in recent international guidelines, which recognize the possibility of participation in recreational and competitive physical activities for selected low-risk patients.

The clinical message proposed by our review is also supported by Albulushi et al. (32). The two studies converge on a central point: physical activity, when structured and supervised, appears to be generally safe in patients with HCM and is associated with functional and quality of life benefits. The common idea is to move away from generalized prohibitions and towards personalized prescriptions, with risk stratification, monitoring, and gradual progression of intensity. For both studies, gray areas remain, such as the optimal dose, the role of high intensity, the duration needed to consolidate benefits, and management in carriers of ICDs or in the presence of extensive fibrosis.

Despite increasing evidence supporting exercise in HCM, there remain no clear, harmonized recommendations for prescribing physical activity in patients with obstructive hypertrophic cardiomyopathy. Optimal exercise modalities, safe intensity thresholds, and contraindications for this subgroup remain areas of uncertainty. Dedicated studies focusing on obstructive phenotypes are urgently needed.

## Conclusions

The meta-analysis included 8 studies on 2217 patients with HCM, of whom 1204 were in the exercise group and 1013 in the control group.

In summary, this meta-analysis provides encouraging evidence that structured and supervised exercise appears safe and improves functional capacity in patients with hypertrophic cardiomyopathy, particularly in those classified as low-risk. However, these conclusions must remain conditional given the limited follow-up duration, variability in exercise protocols, and risk of bias in several included studies. Exercise prescription should therefore be individualized, guided by specialist evaluation, and not extrapolated to vigorous or competitive sports participation without further evidence.

### Study limitations

The main limitations of this meta-analysis are as follows:

The overall risk of bias was substantial across multiple domains, affecting both randomized control trials (RCTs) and observational studies. In particular, limitations in randomized procedures, blinding and outcome assessment may have introduced systematic error and influenced effect estimates.

Another important limitation concerns the small sample sizes of most available studies. Except for the large observational cohort by Lampert et al. (20), the remaining trials and cohort studies enrolled relatively few participants, which reduces statistical power and widens confidence intervals, particularly for rare outcomes such as arrhythmic events or sudden cardiac death. This limitation restricts the generalizability of the safety conclusions, especially regarding vigorous-intensity activities.

The short duration of follow-up limits the ability to evaluate long-term safety and sustainability of benefits. RCTs with follow-up periods of only 12–24 weeks cannot adequately capture the persistence of physiological adaptations, potential disease progression or hard clinical endpoints such as mortality and MACE.

Finally, the absence of standardized exercise interventions led to considerable heterogeneity in training modality, intensity, frequency, session duration and supervision. This variability hampers the identification of an optimal exercise prescription and reduces the generalizability of the findings to clinical practice.

### Future research directions

The results of this meta-analysis highlight multiple perspectives for future research in the field of exercise for patients with hypertrophic cardiomyopathy. Future studies should focus on conducting adequately powered RCTs with larger sample sizes and extended follow-up durations to assess the long-term effects of exercise on disease progression, quality of life, and cardiovascular prognosis. Research should investigate the efficacy of different types of training (aerobic, resistance, combined) in specific subgroups of patients.

In addition, future research should aim to develop precision-based algorithms for exercise prescription that integrate genetic, phenotype and functional data, thereby enabling truly individualized exercise programs aligned with each patient's physiological and clinical profile.

The studies included in this meta-analysis primarily assessed functional outcomes (VO₂ peak) and clinical safety events (MACE). However, they did not systematically evaluate disease progression markers such as left ventricular wall thickness, extent of late gadolinium enhancement, atrial remodeling, or arrhythmic burden during follow-up. To determine whether vigorous or competitive-level exercise may influence the natural history of HCM, future studies should incorporate longitudinal imaging and electrophysiological endpoints.
